# α-amanitin resistance in *Drosophila melanogaster*: A genome-wide association approach

**DOI:** 10.1371/journal.pone.0173162

**Published:** 2017-02-27

**Authors:** Chelsea L. Mitchell, Catrina E. Latuszek, Kara R. Vogel, Ian M. Greenlund, Rebecca E. Hobmeier, Olivia K. Ingram, Shannon R. Dufek, Jared L. Pecore, Felicia R. Nip, Zachary J. Johnson, Xiaohui Ji, Hairong Wei, Oliver Gailing, Thomas Werner

**Affiliations:** 1 Department of Biological Sciences, Michigan Technological University, 1400 Townsend Dr., Houghton, MI, United States of America; 2 Department of Neurology, University of Wisconsin School of Medicine and Public Health, 1300 University Ave., Madison, WI, United States of America; 3 College of Human Medicine, Michigan State University, Clinical Center, East Lansing, MI, United States of America; 4 U.S. Forest Service, Salt Lake Ranger District 6944 S, 3000 E, Salt Lake City, UT, United States of America; 5 School of Forest Resources and Environmental Sciences, Michigan Technological University, 1400 Townsend Dr., Houghton, MI, United States of America; University of Mississippi, UNITED STATES

## Abstract

We investigated the mechanisms of mushroom toxin resistance in the *Drosophila* Genetic Reference Panel (DGRP) fly lines, using genome-wide association studies (GWAS). While *Drosophila melanogaster* avoids mushrooms in nature, some lines are surprisingly resistant to α-amanitin—a toxin found solely in mushrooms. This resistance may represent a pre-adaptation, which might enable this species to invade the mushroom niche in the future. Although our previous microarray study had strongly suggested that pesticide-metabolizing detoxification genes confer α-amanitin resistance in a Taiwanese *D*. *melanogaster* line Ama-KTT, none of the traditional detoxification genes were among the top candidate genes resulting from the GWAS in the current study. Instead, we identified *Megalin*, *Tequila*, and *widerborst* as candidate genes underlying the α-amanitin resistance phenotype in the North American DGRP lines, all three of which are connected to the Target of Rapamycin (TOR) pathway. Both *widerborst* and *Tequila* are upstream regulators of TOR, and TOR is a key regulator of autophagy and *Megalin*-mediated endocytosis. We suggest that endocytosis and autophagy of α-amanitin, followed by lysosomal degradation of the toxin, is one of the mechanisms that confer α-amanitin resistance in the DGRP lines.

## Introduction

The mechanisms that allow some drosophilid species to breed in toxic mushrooms have remained largely elusive for the past half century, despite a series of studies attempting to reveal the genetic basis of this phenomenon [1–8]. α-Amanitin is the principal toxin of several deadly poisonous mushroom species, including the death cap (*Amanita phalloides*) and destroying angel (*Amanita bisporigera*, *A*. *ocreata*, and *A*. *virosa*) [9, 10]. This toxin inhibits the function of RNA polymerase II, halting all eukaryotic messenger RNA production [11]. Among eukaryotes, only a few species, such as *Drosophila guttifera*, *D*. *falleni* and *D*. *recens* [1, 3, 5, 8, 12], are adapted to breeding in and feeding on these very poisonous mushrooms. Thus, there is virtually no competition for this food source [3, 5, 8, 12], and breeding in these mushroom species provides protection against parasitic nematodes, which are susceptible to mushroom toxins [13, 14].

Typically, *D*. *melanogaster* oviposits in and feeds on fermenting fruit and strictly avoids mushrooms. Although α-amanitin is solely produced by mushrooms, a few lines of *D*. *melanogaster* were shown to be up to two orders of magnitude more resistant to α-amanitin than susceptible lines of this species. Resistant lines can tolerate up to ~ 10 μg of α-amanitin per gram of food, as compared to ~ 0.1 μg/g in very susceptible lines [2, 7, 15, 16]. In contrast, mycophagous species develop in mushrooms containing up to 1200 μg of total α-amanitin [17] and are deleteriously affected by α-amanitin in the range of 750 to 1000 μg/g [5]. For comparison, the human LC_50_ is 0.1 μg/g body weight, which can make the consumption of a single mushroom lethal [17]. Because *D*. *melanogaster* has no known history of mushroom-feeding in the wild, and the resistance is insufficient to safely consume highly toxic mushrooms, α-amanitin resistance may be a pre-adaptation for a possible mushroom niche invasion in the future.

In our previous microarray study [15], we identified four possible molecular mechanisms for α-amanitin resistance in a *D*. *melanogaster* line from Taiwan (Ama-KTT, collected in 1968 in Kenting): 1) blockage by cuticular proteins, 2) detoxification by phase I and II detoxification enzymes: Cytochrome P450s (P450s), Glutathione-S-transferases (GSTs), and UDP glucuronosyl transferases (UGTs), 3) cytoplasmic sequestration in lipid particles, and 4) cleavage by peptidases. The top three candidate genes resulting from that study, *Cyp6a2*, *Cyp12d1-d*, and *Cyp12d1-p*, have all been shown to detoxify pesticides, such as dichlorodiphenyltrichloroethane (DDT), imidacloprid, dicyclanil, and atrazine [18–22]. Thus, pesticides may have originally triggered the evolution of α-amanitin resistance in this Taiwanese fly line.

Here, lines from the *Drosophila* Genetic Reference Panel (DGRP) [23] were tested to determine whether α-amanitin resistance is a variable trait in a North American population of *D*. *melanogaster* and to identify the candidate genes/mechanisms that protect these lines from α-amanitin. We measured the phenotypes as the fraction of larvae emerging as adults on media supplemented with different α-amanitin concentrations. We performed four tests: 1) a GWAS for 180 DGRP lines using a low toxin concentration (0.2 μg/g α-amanitin/food), 2) a GWAS for 180 DGRP lines using a relatively high toxin concentration (2.0 μg/g α-amanitin/food), 3) a GWAS using lethal concentration 50 (LC_50_) values obtained for 37 DGRP lines, for which RNA-seq data exist, and 4) a correlation analysis of larval resistance with adult transcriptome data among the 37 DGRP lines. We show that α-amanitin resistance is a common and variable trait among all tested lines. We identified three genes, *Megalin* (*mgl*), *Tequila* (*teq*), and *widerborst* (*wdb*) that may interact with the Target of Rapamycin (TOR) pathway to mediate α-amanitin resistance through endocytic and autophagic sequestration and degradation.

## Results and discussion

### α-amanitin resistance variation and trait definition

**Fig 1:**
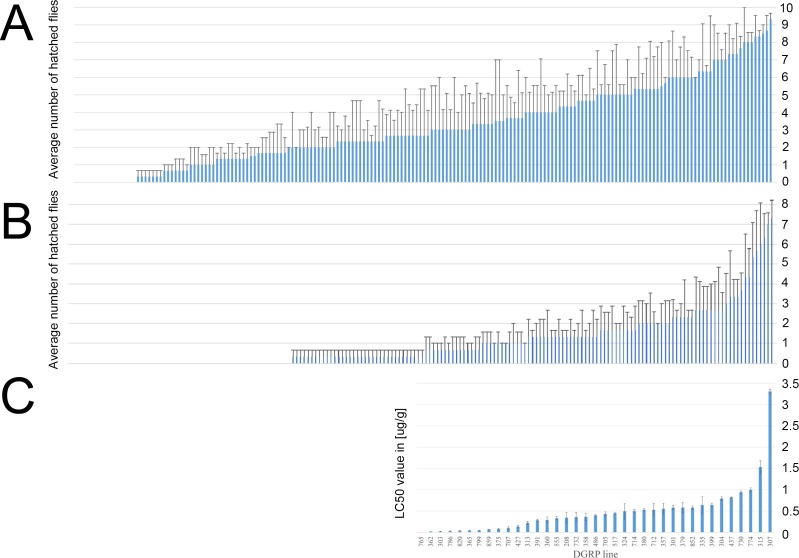
Larval viability variation in the DGRP lines in response to α-amanitin. The y-axis shows individual viability values, while the x-axis represents the individual DGRP lines. The lines are sorted from lowest α-amanitin resistance (left) to highest α-amanitin resistance (right). The error bars represent the standard error of the mean (SEM). A) 180 lines tested on 0.2 μg/g α-amanitin. (Individual line numbers are not shown but can be found in S1 Table). The y-axis represents the average number of flies hatched from 10 larvae placed on toxic food. B) 180 lines tested on 2.0 μg/g α-amanitin. (Individual line numbers are not shown but can be can be found in S1 Table). The y-axis represents the average hatch counts out of 10 larvae placed on toxic food. C). The y-axis represents the LC_50_ values of the 37-line subset. The line numbers are shown on the x-axis.

We first tested if the *D*. *melanogaster* DGRP lines varied in their resistance to α-amanitin, using two α-amanitin concentrations (0.2 μg/g and 2.0 μg/g) that we predicted to allow for an evaluation of the resistance variation among all DGRP lines. We placed ten freshly hatched first-instar larvae per fly line and toxin concentration into vials and performed three replicates for a total of 30 larvae per line and concentration. We defined the phenotypic value for this experiment as the average number of adult flies emerging from the three replicate vials, ranging from 0–10 flies. The data are shown in Fig 1A and 1B. Out of the 189 DGRP lines initially tested, twelve lines displayed relatively high resistance to α-amanitin on 2.0 μg/g, 83 showed intermediate resistance, 91 showed virtually no resistance on 2.0 μg/g, while three lines did not produce enough eggs for the experiments. Based on these data (S1 Table), we concluded that α-amanitin resistance in the DGRP lines is a quantitative trait amenable to GWAS.

Following this experiment, we calculated the LC_50_ values of a 37-line subset of these 180 lines. Because α-amanitin is very expensive and LC_50_ measurements are extremely labor-intensive and time-consuming, we limited the determination of the LC_50_ values to those 37 DGRP lines, for which adult transcriptome data are available [24] to conduct later LC_50_/gene expression correlation tests. To calculate the LC_50_ values, we tested each of the 37 lines on seven α-amanitin concentrations, including controls lacking α-amanitin. For each line and concentration, ten internal replicates of ten first-instar larvae per feeding tube were performed, and each experiment was performed three times. Thus, we tested a total of 300 larvae per concentration and fly line, as compared to only 30 larvae per concentration in the 180-line experiment on 0.2 and 2.0 μg/g. The resulting LC_50_ values were defined as the phenotypic trait for this experiment and are shown in Fig 1C and S2 Table.

We note that among the 37-line subset, the resistance data from the 2 μg/g α-amanitin treatment and the LC_50_ experiment were only weakly correlated (r = 0.24, p = 0.075, linear regression analysis) (S3 Table). This low correlation was caused by at least two factors. First, the 2 μg/g α-amanitin experiment data had a 10 X lower sample size than the LC_50_ calculation, as mentioned earlier. Second, the two lines with the highest LC_50_ values (lines DGRP-307 and 315) did not show a very high viability in the 2 μg/g α-amanitin experiment. Although the initial association between both data sets was weak, it increased when these two lines with very high LC_50_ values were excluded from the comparison (r = 0.39, p = 0.02) (S4 Table). Among the 37 lines, the resistance data at 0.2 μg/g α-amanitin were not associated with the LC_50_ values (r = 0.17, p = 0.31), and the association remained non-significant after exclusion of the two lines with very high LC_50_ values (r = 0.32, p = 0.06). Furthermore, the resistance data among all 180 lines at 2 μg/g α-amanitin and 0.2 μg/g α-amanitin revealed a weak and barely significant correlation (r = 0.13, p = 0.037) (S3 Table). In order to test to what degree viability associated with the α-amanitin resistance phenotype is genetically determined, we calculated the broad-sense heritability values. They were 0.95 for the 37-line LC_50_ data, 0.46 for the 180-line 2.0 μg/g data, and 0.32 for the 180-line 0.2 μg/g data. Thus, the 180-line 0.2 μg/g data contain the greatest level of random mortality that is not associated with the α-amanitin-resistance phenotype.

### The 180-line GWAS on 2.0 μg/g versus the 37-line GWAS

**Fig 2:**
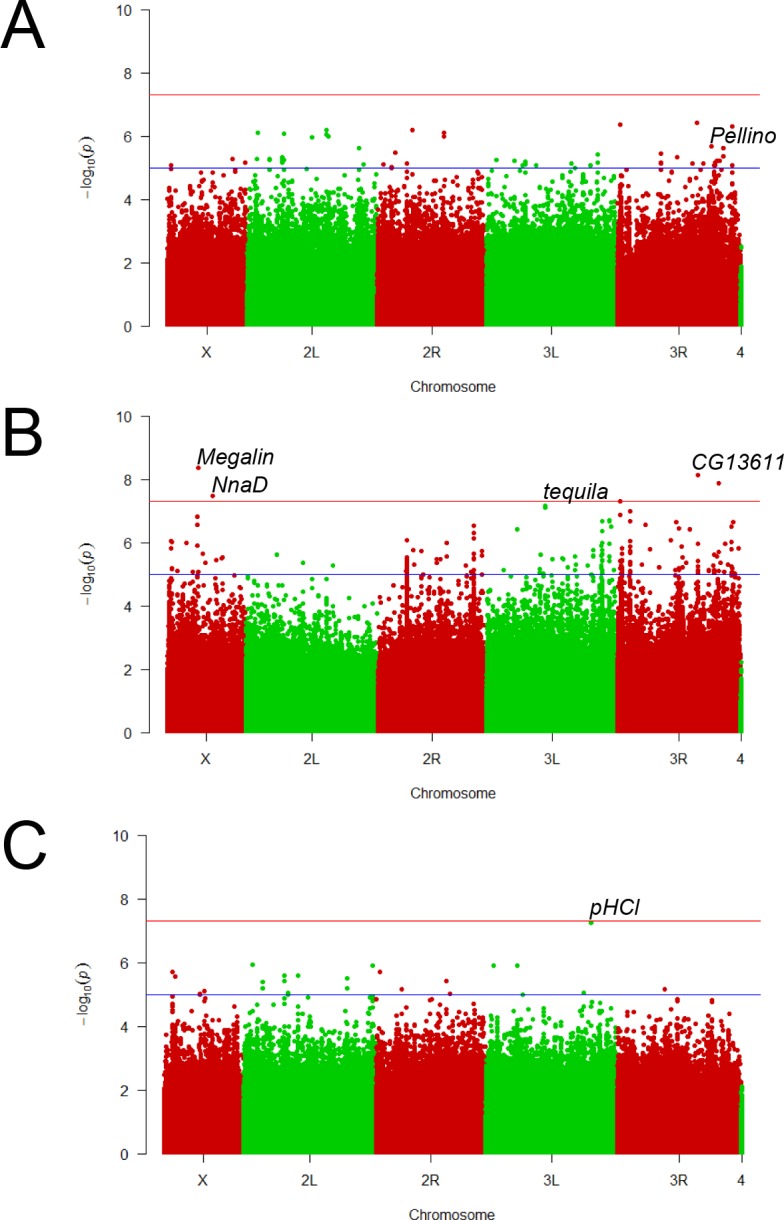
Manhattan plots for the three GWAS. A) 37-line GWAS using LC_50_ values, B) 180-line GWAS on 2.0 μg/g α-amanitin, C) 180-line GWAS on 0.2 μg/g α-amanitin. Selected significant gene names are printed on the top right of the corresponding dots in the graphs.

After establishing that α-amanitin resistance varies among the DGRP lines, we next performed three separate GWAS to identify candidate genes underlying α-amanitin resistance in the DGRP lines: 1) a 37-line GWAS using the LC_50_ values, 2) a 180-line GWAS on 2.0 μg/g α-amanitin, and 3) a 180-line GWAS on 0.2 μg/g α-amanitin. The phenotypic values associated with each line were submitted to the DGRP website at dgrp2.gnets.ncsu.edu for analysis. The Manhattan plots for all three GWAS are shown in Fig 2. The results for the 37-line GWAS are provided in S5 Table, the results for the 180-line GWAS on 2.0 μg/g α-amanitin are listed in S6 Table, and the 180-line GWAS results on 0.2 μg/g α-amanitin can be found in S7 Table.

Because the 180-line data on 0.2 μg/g showed the lowest heritability value, we will first discuss the 37-line GWAS results using LC_50_ values and the 180-line GWAS on 2.0 μg/g. Table 1 summarizes the top 11 candidate genes from S5 and S6 Tables, which fulfilled at least one of three requirements: 1) the single mixed p-value was below the Bonferroni p-value cutoff of < 2.60E-08 (“strict”), 2) the single mixed p-value was above but still close to the Bonferroni cutoff value (“relaxed”, <1.00E-05), and/or 3) the candidate genes were identified in both GWAS datasets, for one of which the smallest p-value was at least “relaxed” (<1.00E-05).

**Table 1:** Candidate genes resulting from the 180-line GWAS on 2.0 μg/g and the 37-line GWAS.

Candidate gene symbol	180-line GWAS on 2.0 μg/g: lowest single mixed p-value	37-line GWAS with LC_50_ values: lowest single mixed p-value	180-line GWAS on 2.0 μg/g: lowest FDR (BH) p-value	37-line GWAS with LC_50_ values: lowest FDR (BH) p-value	Cyto. map location/chromosome arm	Selected known or predicted functions, as per FlyBase [25]
*mgl*	4.26E-09 ***	9.02E-04 *	**0.00734**	0.41622	8D10 / X	LDL receptor class A, chitin-based cuticle development, endocytic receptor in epithelial cells
*CG13611*	7.66E-09 ***	N/A	**0.00734**	N/A	95F8-95F9 / 3R	Oxidoreductase activity
*NnaD*	3.45E-08 **	N/A	**0.01478**	N/A	12B4 / X	Peptidase M14, carboxypeptidase A
*teq*	6.91E-08 **	N/A	**0.01478**	N/A	66F4-66F5 / 3L	LDL receptor class A, scavenger receptor activity; chitin binding; serine-type endopeptidase activity
*Scr*	2.30E-06 **	3.54E-06 **	0.06678	0.24164	84A5 / 3R	Midgut development, transcription factor activity
*Pli*	1.99E-05 *	3.98E-07 **	0.13086	0.09639	95C5-95C8 / 3R	Immune response
*Rbp6*	1.69E-04 *	3.82E-06 **	0.28190	0.13828	73E5-74A1 / 3L	mRNA-binding, stem cell development
*Hs6st*	5.39E-05 *	4.71E-06 **	0.18521	0.13828	92B8-92C1 / 3R	Sulfotransferase activity
*Ets65A*	7.47E-04 *	6.65E-06 **	0.45526	0.13828	65A6 / 3L	Regulation of transcription
*wdb*	4.59E-04 *	7.20E-06 **	0.39087	0.13828	98A6-98A8 / 3R	Protein phosphatase 2A
*sti*	5.40E-04 *	7.27E-06 **	0.40926	0.13828	69C4 / 3L	Threonine kinase activity

The single mixed p-values are.

*** = strict (below the Bonferroni cutoff value of 2.60E-08)

** = relaxed (between the Bonferroni cutoff value and 1.00E-05), or

* = suggestive (between 1.00E-04 and 9.99E-04).

N/A = these SNPs do not segregate in the 37-line GWAS. We also provide a multiple test correction from the entire set of 1.9 million GWAS tests for each phenotype and calculated the False Discovery Rate from the single mixed p-values: FDR (BH) = Benjamini Hochberg False Discovery Rate (bold values are significant).

The 180-line GWAS on 0.2 μg/g resulted in one gene with a very low p-value. This gene, *pHCl*, showed a single-mixed p-value of 5.60E-08 (FDR (BH) p = 0.05423)). It encodes a neurotransmitter-gated ion channel transmembrane protein [25]. However, none of the genes identified in the 180-line GWAS on 0.2 μg/g had known functions that link them to possible toxin resistance mechanisms (S7 Table). As discussed earlier, the reason for this lack of suitable candidate genes may be random mortality at the larval stage that is not associated with the α-amanitin resistance phenotype. We note that some DGRP lines, including resistant ones, showed poor viability on the low 0.2 μg/g toxin concentration, while they survived better on higher α-amanitin concentrations. Although the reason for this paradox is unknown, it has been observed in two unrelated α-amanitin-resistant lines of *D*. *melanogaster* [2, 3], as well as in several mycophagous species [3, 8].

### Discussion of the top eleven candidate genes

*Megalin* (*mgl*) was the most significant candidate gene. We identified this gene in both the 180-line GWAS on 2.0 μg/g and the 37-line GWAS. In the 180-line GWAS on 2.0 μg/g, *mgl* was represented by one intronic single nucleotide polymorphism (SNP) (X_9293649_SNP) with a "strict" p-value of p = 4.26E-09. Additionally, we found two "suggestive" intronic *mgl* SNPs in the 37-line GWAS (X_9355099_SNP and X_9355132_SNP, p = 9.02E-04 each). We speculate that intronic SNPs may alter *cis*-regulatory modules, and that higher *mgl* gene expression may confer higher resistance to α-amanitin. *Megalin* encodes a conserved endocytosis and transcytosis receptor found in the apical region of many epithelial cell types in both vertebrates and invertebrates [26–31]. *Megalin* is a member of the low-density lipoprotein receptor (LDLR) family and has more than 40 different protein ligands (including toxic substances [32]), which are degraded in the lysosome upon receptor binding and endocytosis [26, 27, 33]. Endocytosis is a process known to be regulated in the epithelium and fat body of *Drosophila* by TOR [34]. Furthermore, *Megalin* endocytosis is induced by the Peroxisome Proliferator-Activated Receptor (PPAR), a protein with the capacity to increase autophagy (also see *wdb*, below) and a repressor of TOR [35]. If *Megalin* confers α-amanitin resistance, as suggested by our GWAS results, a possible scenario would be the uptake/sequestration of α-amanitin by the Megalin protein and the subsequent degradation by endocytic processes up-regulated in the midgut epithelium, where the food is absorbed in *Drosophila* larvae.

**Fig 3:**
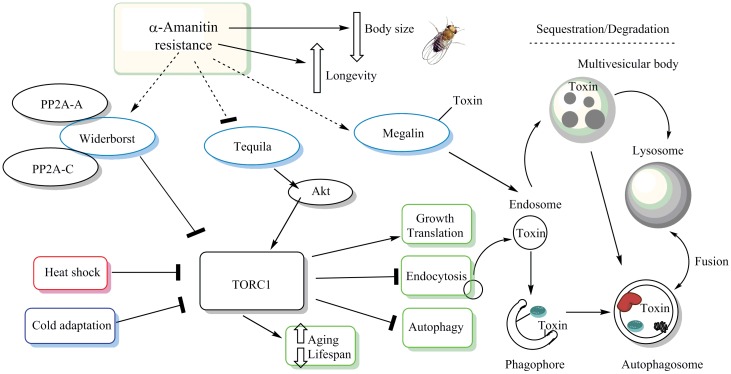
The TOR pathway may be central to the evolution of α-amanitin resistance in the DGRP lines. The schematic represents a suggested model for how α-amanitin resistance interplays with the TOR pathway via three of the top candidate genes suggested by our GWAS: *widerborst*, *Tequila*, and *Megalin*. The proteins Widerborst and Tequila are known upstream regulators of TOR, influencing autophagy. TOR is a critical repressor of autophagy and *Megalin*-mediated endocytosis. Both the endocytic and autophagic catabolic processes end with the degradation and recycling of macromolecules in lysosomes. We hypothesize that in α-amanitin-resistant flies, Widerborst protein levels are up-regulated, while Tequila protein levels are down-regulated to collectively repress TOR, through heterotrimeric PP2A-A-Wbd-PP2A-C/TOR and Akt/TOR interactions, respectively. Megalin protein is hypothesized to be up-regulated and to sequester α-amanitin to the endosome. TOR inactivation may play a role in the elimination of cytoplasmic α-amanitin by de-repression of the autophagic process. As a result, the toxin would become entrapped in an elongating phagophore, and the autophagosome would then undergo lysosomal fusion, followed by degradation of α-amanitin. Down-regulation of TOR in α-amanitin-resistant flies is also consistent with the reduced thorax size (in the presence of α-amanitin) and increased longevity observed in α-amanitin resistant flies [16].

The *Tequila* (*teq*) gene was represented by five intronic SNPs and indels with "relaxed" p-values in the 180-line GWAS on 2.0 μg/g: 3L_9069423_SNP (p = 6.91E-08), 3L_9069400_SNP (p = 7.15E-08), 3L_9069410_INS (p = 7.15E-08), 3L_9069403_SNP (p = 7.38E-08), and 3L_9069415_SNP (p = 7.71E-08). Interestingly, *teq* activation by insulin signaling has been shown to increase Akt activity, a key modulator of TOR signaling that can activate TOR complex 1 (TORC1) [36]. We hypothesize that the intronic SNPs in the *teq* locus cause a decrease in *teq* gene expression, which would have a repressing effect on TOR. Previous studies in *Drosophila* have associated *teq* silencing with increased lifespan [36] and long-term memory formation [37]. Our previous work also correlated α-amanitin resistance to increased lifespan and reduction of body size; both could potentially be explained by reduced TOR activity [16] (Fig 3). We note that both *Megalin* and *Tequila* had highly significant FDR (BH) values of 0.00734 and 0.01478, respectively.

The *widerborst* (*wdb*) gene was represented by four intronic SNPs with "relaxed" p-values in our 37-line GWAS results: 3R_23395889_SNP, 3R_23395891_SNP, 3R_23395908_SNP (p = 7.20E-06 each), and 3R_23395900_SNP (p = 8.24E-06). Also, several SNPs of mostly intronic nature were identified in this GWAS with "suggestive" p-values. Additionally, we identified one intronic SNP with a "suggestive" p-value in 180-line GWAS on 2.0 μg/g. *Widerborst* is a key regulator of the catabolic process of autophagy and has also been shown to have a lifespan-increasing effect in *Drosophila* [38]. Current knowledge indicates that common machinery contributes to the regulation and mechanisms involved in degradation by the endosomal and autophagic pathways [28]. For example, TORC1 regulation and the early and recycling endosomes of the endocytic pathway contribute to phagophore formation and maturation of the phagolysosome [28] (Fig 3). The Ser/Thr Phosphatase Protein Phosphatase 2A (PP2A) regulatory subunit Widerborst inhibits signaling upstream of TOR via the heterotrimeric holoenzyme PP2A-A/Wdb/PP2A-C complex (Fig 3). The Widerborst protein acts upstream to inhibit TOR via a genetic interaction with PtdIns3K/PTEN/Akt [39]. Thus, our results and previous studies cumulatively suggest that a TOR-inhibiting mechanism is involved in α-amanitin resistance.

*Ets at 65A* (*Ets65A*) is a transcription factor gene of largely unknown function. *Ets65A* is situated at the same chromosomal position to which α-amanitin resistance has been mapped in two previous studies [2, 7], making this factor particularly interesting. Begun and Whitley (2000) [7] suggested that *Mdr65* may be the α-amanitin resistance-conferring gene at that locus. In the 37-line GWAS, we identified two *Ets65A* SNPs with "relaxed" p-values: 3L_6097046_SNP (p = 6.65E-06, intronic) and 3L_6096774_SNP (p = 7.89E-06, synonymous coding). In the 180-line GWAS on 2.0 μg/g, we found one intronic SNP of *Ets65A* with a "suggestive" p-value. The *Ets65A* gene is expressed in adipose tissue [40] and associated with cold adaptation [41] and olfactory behavior [42] in *D*. *melanogaster*. It is worth noting that the TOR pathway not only responds to cellular energy cues, but has also been shown to play a critical role in heat shock and cold-induced stress responses [43]. However, *Ets65A* has not been shown to mediate any detoxification responses yet.

*Sex combs reduced* (*Scr*) encodes a transcription factor involved in anterior midgut development in *Drosophila* embryos [44, 45]. We identified four intronic *Scr* SNPs with “relaxed” p-values in our 180-line GWAS on 2.0 μg/g: 3R_2658997_SNP (p = 2.30E-06), 3R_2668803_SNP (p = 3.54E-06), 3R_2672660_SNP (p = 7.12E-06), and 3R_2665283_MNP (p = 9.41E-06). The 37-line GWAS uncovered seven additional SNPs for *Scr* with "suggestive" p-values, five of which were present in the 3' UTR, and the other two were located in introns. Because food absorption happens in the midgut, this organ is a strategic place for mushroom toxin resistance mechanisms to be deployed.

*CG13611* is a gene with only predicted functions. We identified it in the 180-line GWAS on 2.0 μg/g as the second of two genes that show at least one SNP with a "strict" p-value. *CG13611* was represented by three SNPs: 3R_20106448_SNP (p = 7.66E-09, non-synonymous coding), 3R_20106547_SNP (p = 1.08E-06, non-synonymous coding), and 3R_20105165_SNP (p = 6.11E-05, upstream). The CG13611 protein is predicted to have oxidoreductase activity [25]. We have previously shown that oxidoreductase activity may be an important process to help detoxify α-amanitin [15]. It may thus be possible that CG13611 exerts a function similar or identical to phase I detoxification. The observed non-synonymous coding changes might alter the enzyme activity, possibly leading to differences in the enzyme's ability to chemically modify α-amanitin. We note that *CG13611* had a highly significant FDR (BH) p-value of 0.00734.

*Pellino* (*Pli*) was identified as the top candidate gene in the 37-line GWAS, represented by a single intronic SNP (3R_19707888_SNP, p = 3.98E-07). We further found five additional intronic SNPs in the 180-line GWAS on 2.0 μg/g with "suggestive" p-values. The *Pli* gene encodes an intracellular, positive regulator of the innate immune response in animals ranging from insects to man [46]. The Pellino protein regulates the Toll/Toll-like pathways, which respond to microbial antigens [47–49]. Interestingly, human Pellino-1 has been shown to confer resistance to two chemotherapeutic drugs in lung cancer cells [50]. Although it is not entirely clear how an innate immune pathway might lead to α-amanitin resistance in fruit flies, the innate immune system is capable of identifying and removing foreign substances from the body, and links between the innate immunity and autophagy have been described earlier [51, 52].

*NnaD* was represented by one SNP in the 180-line GWAS on 2.0 μg/g: X_13585381_SNP (p = 3.45E-08), which is located upstream of the *NnaD* gene. Its protein function is described as peptidase M14 and carboxypeptidase A [25]. The mouse orthologue protein Nna1 is known to hydrolyze polyglutamate-containing substrates in neurons [53]. Because α-amanitin is a peptide, we speculate that NnaD may be involved in the proteolytic degradation of α-amanitin.

We do not currently offer an explanation for the possible roles of *RNA-binding protein 6* (*Rbp6*), *Heparan sulfate 6-O-sulfotransferase* (*Hs6st*), and *sticky* (*sti*) in the detoxification of α-amanitin. All three genes showed SNPs with "relaxed" p-values in the 37-line GWAS and SNPs with "suggestive" p-values in the 180-line GWAS on 2.0 μg/g.

### The Pearson correlation analysis: Comparing larval resistance to the adult transcriptome

We decided to focus on the 37-line subset because their adult transcriptomes were sequenced along with their genomes. We performed a Pearson correlation analysis, using our larval LC_50_ values and the publicly available RNA-seq data of untreated male and female adults. As a result, we identified the *Translocator protein* (*Tspo*) gene (S8 Table) with negative correlation coefficients in females of -0.72 (p = 6.35E-07) and males -0.67 (p = 2.00E-05). The female p-value was below the Bonferroni cut-off for this analysis (p = 4.95E-06), while the male correlation p-value was above the cutoff value. We also calculated the FDR-corrected p-values for the genes in this analysis and found 187 genes with corrected p-values <0.05. We were unable to link the identified genes to toxin resistance. *Tspo* was the only gene resulting from the Pearson analysis that was also identified in one of our two GWAS. This gene was highly significant (corrected p = 0.00160 in females and = 0.01686 in males). In the 180-line GWAS on 2.0 μg/g, two SNPs with "suggestive" p-values were identified: one in the 3' UTR and one forming a synonymous stop codon (AGG to AGA). *Tspo* encodes an outer mitochondrial membrane protein. Studies in *Tspo* mutants revealed important roles for this gene in lifespan determination, oxidative phosphorylation, positive regulation of apoptosis, control of ethanol-mediated behavior, as well as neurodegeneration [54, 55].

### Different genes and mechanisms may underlie α-amanitin resistance in different populations and species

The rationale of using *D*. *melanogaster* to study mushroom toxin resistance is that *D*. *melanogaster* is currently our best genetic *Drosophila* model organism, and that there are no sequenced lines available for mycophagous species to allow for GWAS analyses. Studies in *D*. *melanogaster* have thus far suggested six possible mechanisms to evolve resistance to α-amanitin: 1) In a lab mutagenesis screen, an RNA-polymerase II mutant was identified that made the line 250-fold more resistant to α-amanitin than wild type [56]. 2) Our own laboratory has previously shown that the Taiwanese *D*. *melanogaster* line Ama-KTT [15] may have evolved resistance through 2) phase I detoxification (mediated by *Cyp* genes), 3) phase II detoxification (mediated by *Gst* and *Ugt* genes), 4) sequestration of α-amanitin in lipid particles, and 5) peptidase cleavage. Interestingly, the CYP450 inhibitor piperonyl butoxide (PBO) caused a dramatic reduction in α-amanitin resistance in the mycophagous species *D*. *phalerata* [8], suggesting that *Cyp* genes may play a role even in some mycophagous species. 6) In the current study, we show evidence that the TOR pathway may play a role in mediating the degradation of α-amanitin through endocytosis and autophagy. It is possible that mycophagous *Drosophila* species use some or a combination of the mechanisms that we have found in *D*. *melanogaster* to gain resistance to α-amanitin.

Why *D*. *melanogaster* has evolved α-amanitin resistance is not clear. At least in the case of the Taiwanese line Ama-KTT, pesticide exposure may have triggered the up-regulation of detoxification enzymes, which may display cross-resistance to α-amanitin [15]. We speculate that the evolution of α-amanitin resistance is a pre-adaptation, preparing the species to invade the mushroom niche in the future. Considering that *D*. *melanogaster* can complete its development on non-toxic fungi in the laboratory, such as Baker's yeast, a niche expansion to include other fungi seems plausible. Such a niche invasion may be aided by our previous observation that females of resistant strains show a higher fecundity when they grew up on food containing sublethal α-amanitin concentrations [16]. This increased fecundity might help the species to adapt to a diet of increasingly toxic mushrooms.

### Limitations

Although it would be ideal to establish highly accurate LC_50_ values for all 180 DGRP lines, it would be very time-consuming and take many resources to set up such an experiment. Working with α-amanitin requires a tradeoff between the number of fly lines used and the accuracy of the phenotypic values to measure. For our 180-line GWAS on 2.0 μg/g and on 0.2 μg/g, we had to accept a relatively low accuracy of the resistance measurement (30 larvae per line). In the 37-line GWAS, we were able to use highly accurate phenotypic data (2100 larvae per line to establish the LC_50_ values). We note that the average fly hatch numbers of 3 X 10 larvae used as a trait for the 180-line GWAS on 2.0 and on 0.2 μg/g were not a great predictor of the LC_50_ values among the 37 lines.

Although the main reason for using the 37-line subset of DGRP lines was to discover links between larval α-amanitin resistance and constitutive gene expression differences in adults, we did not identify many meaningful candidate genes by correlating our larval LC_50_ values with the publicly available RNA-seq data of untreated male and female adults, which may have several explanations: 1) larval and adult resistance mechanisms differ from one another, 2) adults of lines that show larval resistance are not α-amanitin-resistant, 3) the resistance mechanism(s) cannot be detected at the transcriptome level in untreated adults, and/or 4) the low number of 37 lines may have limited the chance to identify highly significant associations.

### Future research

Based on the known interplay of three of our eleven top candidate genes (Fig 3), the next line of experiments should focus on the *mgl*, *teq*, and *wdb* genes to test if changes in endocytosis, autophagy, and TOR signaling cause the observed variation in α-amanitin resistance. In order to obtain functional data, gene overexpression experiments, CRISPR-Cas9 knockout, and/or RNAi should be employed to manipulate susceptible lines to become more resistant and resistant lines to become less resistant than they were before the genetic manipulation.

## Conclusions

Our data generated an interesting hypothesis that endocytosis and autophagy may mediate α-amanitin resistance in *D*. *melanogaster*, possibly through the involvement of the TOR pathway. We found that genes identified in our previous microarray study did not show genome-wide significance in the current study. Thus, α-amanitin resistance may have evolved independently in the Taiwanese and the North American populations, utilizing different genes and molecular mechanisms.

## Materials and methods

### Fly lines

We obtained all 189 *D*. *melanogaster* DGRP lines that were publicly available in April of 2012 from the Bloomington *Drosophila* Stock Center at Indiana University. The fly lines were maintained at room temperature at ~ 50% humidity on standard food containing cornmeal, granulated sugar, Brewer’s yeast, agar, and methylparaben as an antifungal agent. Baker’s yeast sprinkles were added to new bottles and vials just before the flies were transferred to them.

### Quantification of larval α-amanitin resistance for the two 180-line GWAS

Four- to six-day-old adult flies were allowed to lay eggs on molasses agar caps that contained fresh Baker’s yeast paste, while being housed in incubators at 25°C, 70% humidity, and a 12:12 hour light/dark cycle. Freshly hatched first-instar larvae were then transferred in groups of ten into 2-mL plastic tubes (USA Scientific), each containing 125 mg of dry instant *Drosophila* food (Carolina) mixed with 375 μL water that contained either 0.1 or 1.0 μg of dissolved α-amanitin, resulting in 500 mg of hydrated food of the concentrations 0.2 or 2.0 μg/g of α-amanitin, respectively. For each DGRP line (including the 37-line subset lines), three such replicate vials were seeded with 10 larvae, and the average number of adult flies hatching from the three replicates was used as the phenotypic value for the two 180-line GWAS.

### Dose-response studies to quantify larval α-amanitin resistance for the 37-line GWAS

Before calculating the LC_50_ values of the 37 lines, we pre-evaluated the α-amanitin resistance of each fly line based on the data obtained for 2.0 μg/g and a second α-amanitin concentration, 0.2 μg/g. This estimate of resistance allowed us to roughly sort the 37 lines into three categories: low, medium, and high α-amanitin resistance. For the exact LC_50_ value calculation, adult flies were allowed to lay eggs on molasses agar caps that contained fresh Baker’s yeast paste, while being housed in incubators at 25°C, 70% humidity, and a 12:12 hour light/dark cycle. Freshly hatched first-instar larvae were transferred in groups of ten into 2-mL plastic tubes (USA Scientific), each containing 125 mg of dry instant *Drosophila* food (Carolina) mixed with 375 μL of liquid (either sterile water or dissolved α-amanitin in water), thus resulting in 500 mg of hydrated food. All lines were then tested on seven α-amanitin concentrations, including the control without α-amanitin: low-resistance lines were tested on 0, 0.025, 0.05, 0.075, 0.1, 0.25, and 0.375 μg/g; medium-resistance lines on 0, 0.33, 0.66, 1.0, 1.33, 1.66, and 2.0 μg/g; and high-resistance lines were tested on 0, 0.66, 1.33, 2.0, 2.66, 3.33, and 4.0 μg/g food. Ten tubes were prepared for each toxin concentration and experiment, and each experiment was performed in three replicates. Only flies that completely hatched from their pupal cases were scored, and experiments in which at least 80% of the control flies hatched were used for the analysis. The experiments were then normalized by setting the control fly hatch numbers to 100%, and the LC_50_ values for each fly line were calculated using scatter plots and the logarithmic trendline function in Microsoft Excel.

### GWAS

Out of the original 189 DGRP lines, three lines (DGRP-492, 727, and 894) did not produce enough eggs for experiments, while six lines (DGRP-274, 378, 387, 398, 476, and 554) produced a submission error on the DGRP2 website (they were marked as invalid lines). The remaining 180 lines listed in S1 Table were used in the current study. In order to identify SNPs associated with α-amanitin resistance in larvae, we submitted the average numbers of hatching flies from three replicates on 2.0 μg/g and on 0.2 μg/g α-amanitin (the two 180-line GWAS) and the LC_50_ values of the 37-line study, not sexed, to the DGRP for analysis, using their web portal at dgrp2.gnets.ncsu.edu. Genotype-phenotype associations were calculated by the DGRP team, using a linear mixed model, which accounts for any effects of *Wolbachia* infection, common polymorphic inversions, and cryptic relatedness in the DGRP lines, as described in detail in [57]. Because 1.9 million SNP variants were tested in the GWAS, the genome-wide significant threshold at the 5% significance level was determined after Bonferroni correction for multiple testing [58] as 2.60E-08 (0.05/2,192,980) [59]. However, 1.00E-05 was often used as a "relaxed" threshold in previous DGRP GWAS studies [60, 61]. For comparability with other studies, we report significance at the "strict" (< 2.60E-08) and "relaxed" (<1.00E-05) significance threshold. For candidate genes that were identified in both GWAS, we further report a "suggestive" significance threshold between 1.00E-04 and 9.99E-04. Candidate genes were selected based on the significance levels in the 180-line and the 37-line GWAS. The associations between LC_50_ data with the average number of hatching flies at 2.0 and 0.2 μg/g α-amanitin among the 37 lines were calculated using a linear regression analysis (S3 and S4 Tables). Broad-sense heritability values were estimated as the ratio of the genetic variance and phenotypic variance according to [62].

### RNA expression correlation analysis

In order to test what candidate genes resulting from the 37-line GWAS were dysregulated in adult flies, we used the publicly available RNA-seq data (http://dgrp2.gnets.ncsu.edu/data.html), which were originally published in [24]. We performed a Pearson correlation analysis, using R software (http://www.R-project.org/). Because the larvae used for the LC_50_ experiments were not sexed, we correlated the LC_50_ values yielded from the DGRP lines in our 37-line experiment with both adult male and female RNA expression values yielded from the same lines independently.

## Supporting information

S1 Table Viability analysis of all 180 DGRP lines on two α-amanitin concentrations. All DGRP lines were tested on two α-amanitin concentrations (0.2 μg/g and 2.0 μg/g), using 30 larvae per concentration and fly line (three replicates of 10 larvae each). Twelve lines displayed relatively high, 83 intermediate, and 91 low resistance to α-amanitin. The values are also graphically represented in Fig 1A and 1C.
(XLSX)

S2 Table Viability analysis of the 37-line subset expressed as LC_50_ values derived from seven α-amanitin concentrations. The LC_50_ values of the lines are shown in the first tab, and the raw data used to calculate the LC_50_ values are presented in the second tab of the Excel file. The LC_50_ values are also graphically represented Fig 1C.
(XLSX)

S3 Table Correlation analysis among the 37-line GWAS and the two 180-line GWAS raw data sets on both 2.0 and 0.2 μg/g of α-amanitin. The two lines with the highest LC_50_ values were not excluded in this file.
(XLSX)

S4 Table Correlation analysis among the 37-line GWAS and the two 180-line GWAS raw data sets on both 2.0 and 0.2 μg/g of α-amanitin. The two lines with the highest LC_50_ values were excluded in this file, leading to an improved correlation among the 37-line and the 180-line data on 2.0 μg/g of α-amanitin.
(XLSX)

S5 Table Complete candidate SNP list resulting from the 37-line GWAS. A cutoff single mixed p-value of 1.00E-03 was used.
(XLSX)

S6 Table Complete candidate SNP list resulting from the 180-line GWAS on 2.0 μg/g of α-amanitin. A cutoff single mixed p-value of 1.00E-03 was used.
(XLSX)

S7 Table Complete candidate SNP list resulting from the 180-line GWAS on 0.2 μg/g of α-amanitin. A cutoff single mixed p-value of 1.00E-03 was used.
(XLSX)

S8 Table Pearson correlation analysis. Larval LC_50_ values and the publicly available RNA-seq data of untreated male and female adults were correlated.
(XLSX)
